# Non‐Small Cell Lung Carcinoma Presenting as Isolated Malignant Ascites in the Absence of a Detectable Primary Tumor: A Case Report and Literature Review

**DOI:** 10.1002/ccr3.73158

**Published:** 2026-07-13

**Authors:** Angad Tiwari, Ashish Sharma, Monica Lewkowicz, Hareesha Rishab Bharadwaj, Khabab Abbasher Hussien Mohamed Ahmed, Edgar Naut

**Affiliations:** ^1^ Department of Internal Medicine Maharani Laxmi Bai Medical College Jhansi Uttar Pradesh India; ^2^ University of Connecticut School of Medicine Hartford Connecticut USA; ^3^ Royal Stoke University Hospital, University Hospitals of North Midlands NHS Trust Stoke‐on‐Trent UK; ^4^ Faculty of Medicine University of Khartoum Khartoum Sudan; ^5^ Department of Pathophysiology, School of Medicine St. George's University Grenada West Indies; ^6^ Trinity Health of New England Hartford Connecticut USA

**Keywords:** ascitic fluid cytology, carcinoma of unknown primary, malignant ascites, non‐small cell lung carcinoma, spontaneous bacterial peritonitis

## Abstract

An 89‐year‐old woman presented with abdominal distension and altered mental status. Non‐portal‐hypertensive ascites suggested spontaneous bacterial peritonitis, but ascitic‐fluid cytology with immunoprofiling and circulating tumor‐DNA confirmed non‐small cell lung carcinoma despite no detectable primary tumor, underscoring early consideration of malignancy in unexplained ascites.

## Summary

- Non‐small cell lung carcinoma (NSCLC) may rarely present as isolated malignant ascites without a detectable primary tumor.
- Malignancy should be considered early in elderly patients with unexplained, non‐portal‐hypertensive ascites, and ascitic‐fluid cytology combined with immunohistochemical and molecular profiling can establish the diagnosis when conventional imaging fails to identify a primary lesion.

## 1 Introduction

Lung cancer is the leading cause of cancer‐related mortality worldwide, and non‐small cell lung carcinoma (NSCLC) accounts for approximately 85% of all cases. In the great majority of patients, the primary tumor arises within the lung and is readily demonstrable on cross‐sectional imaging [1].

NSCLC characteristically disseminates to the brain, liver, adrenal glands, and bones, and less frequently to soft tissue, the peritoneum, and the gastrointestinal tract [2]. In a small subset of patients, metastatic disease is detected before—or in the complete absence of—a radiologically identifiable primary lung lesion, a presentation sometimes termed occult‐primary NSCLC [3]. This overlaps with the broader entity of carcinoma of unknown primary (CUP), which accounts for roughly 3% to 5% of all malignancies; the lung is among the tissues of origin most frequently inferred once immunohistochemical and molecular characterization has been undertaken [4].

Malignant ascites as the sentinel manifestation of NSCLC is decidedly uncommon. Peritoneal and gastrointestinal involvement is far more typical of digestive‐tract, ovarian, and breast primaries, and lung carcinoma accounts for only a small fraction of cases of malignant ascites [5]. When a primary lung lesion cannot be localized, the diagnosis is frequently delayed, appropriate systemic therapy is postponed, and survival is correspondingly poorer than in patients with a known primary [6]. Metastatic disease without a demonstrable primary tumor is therefore widely regarded as a marker of aggressive biology and adverse prognosis, with a reported median survival of less than 1 year [7].

Establishing a confident diagnosis in this setting requires an integrated clinical, radiological, cytological, and molecular evaluation [8]. Cytological assessment of effusion or ascitic fluid, supported by immunohistochemical staining and molecular biomarker profiling, can establish the tissue of origin and direct management when conventional imaging fails to reveal a primary tumor [9].

We describe an 89‐year‐old woman with NSCLC and no identifiable primary tumor who presented with several weeks of worsening abdominal distension and altered mentation. Her illness was initially attributed to decompensated congestive heart failure (CHF) and hypervolemic hyponatremia; however, subsequent evaluation demonstrated non–portal‐hypertensive ascites, and only cytological analysis of the ascitic fluid ultimately revealed malignant cells consistent with NSCLC.

## 2 Case History and Examination

An 89‐year‐old woman with no significant past medical history was brought to the emergency department with a 3‐week history of progressive abdominal distension, decreased oral intake, and altered mental status. Her family also reported lower‐limb edema and short‐term memory loss over the same period.

Two weeks before this presentation she had been hospitalized for volume overload and symptomatic hyponatremia, with a serum sodium of 120 mmol/L. At that time her clinical picture was attributed to hypervolemic hyponatremia in the setting of presumed decompensated heart failure; she was managed for volume overload and hyponatremia and discharged once her sodium had improved. She re‐presented when the abdominal distension and confusion recurred and progressed.

On arrival she was afebrile and hemodynamically stable. Abdominal examination revealed moderate distension with shifting dullness consistent with ascites, and there was bilateral pitting edema of the lower limbs; cardiac and respiratory examination was unremarkable. Transthoracic echocardiography demonstrated a preserved left ventricular ejection fraction of 55% to 65% with only grade 1 diastolic dysfunction—findings that were not in keeping with decompensated systolic heart failure and that argued against CHF as the explanation for her recurrent presentation.

## 3 Methods: Differential Diagnosis, Investigations, and Treatment

This case is reported in line with the CARE (CAse REport) guidelines [10].

### 3.1 Differential Diagnosis

The central question was the etiology of new‐onset ascites. Decompensated CHF and hypervolemic hyponatremia were considered first but were excluded: echocardiography showed preserved systolic function, and a serum–ascites albumin gradient (SAAG) of 0.8 g/dL indicated a low‐gradient, non–portal‐hypertensive ascites, effectively arguing against cardiac and cirrhotic causes. Cirrhosis was thought unlikely in the absence of chronic liver disease or clinical stigmata of portal hypertension. The low SAAG, together with the ascitic‐fluid analysis, redirected the differential toward peritoneal infection and malignancy.

### 3.2 Investigations

**FIGURE 1:**
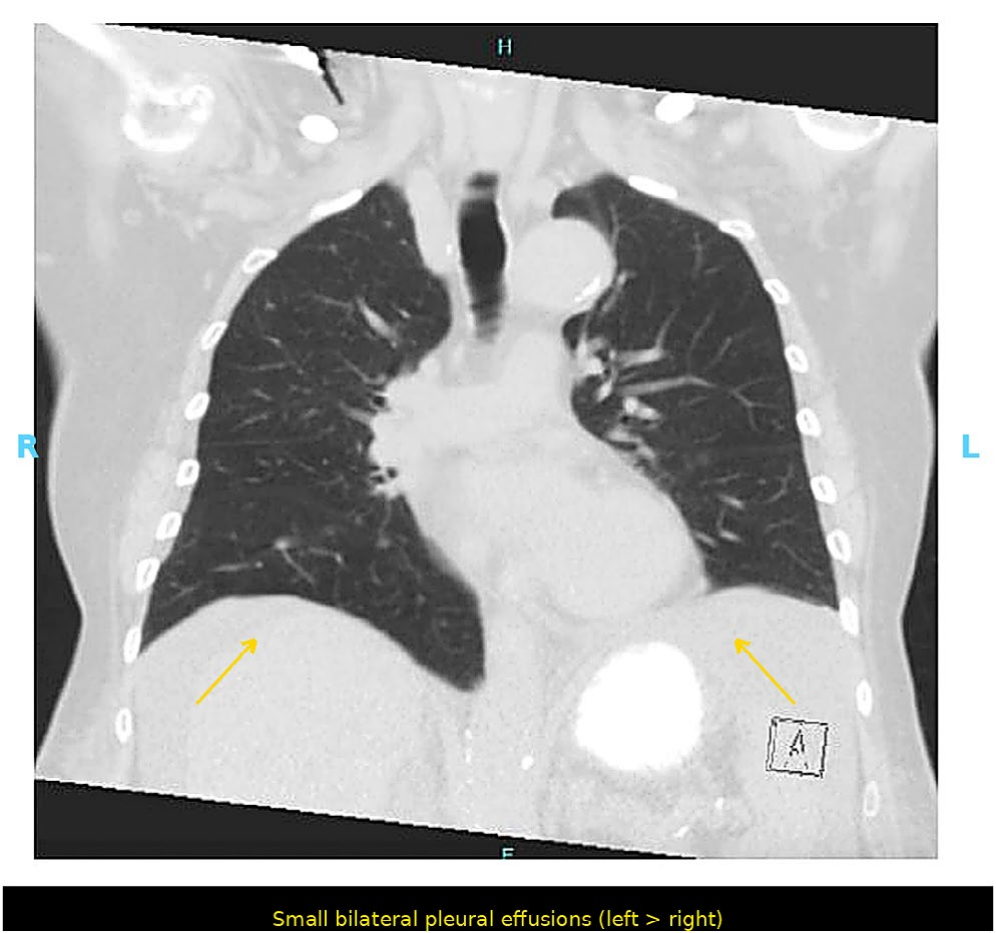
Coronal reformatted image from contrast‐enhanced computed tomography of the chest, displayed in lung windows, at the level of the cardiac chambers and both hemidiaphragms. Small bilateral pleural effusions are present at the costophrenic recesses, larger on the left than on the right (yellow arrows). “R” and “L” denote the patient's right and left sides; “H” and “F” are the scanner‐generated head and feet orientation markers. No primary pulmonary mass is identified.

Contrast‐enhanced computed tomography (CT) of the chest, abdomen, and pelvis demonstrated moderate ascites and small bilateral pleural effusions (left greater than right) but revealed no pulmonary, hepatic, gastrointestinal, gynecological, or other primary tumor (Figure 1). Diagnostic paracentesis yielded fluid containing 1293 white cells/μL with 62% polymorphonuclear neutrophils, meeting the criterion for spontaneous bacterial peritonitis (SBP); ascitic cultures were negative (Table 1). After nephrology review, the hyponatremia was reattributed to the syndrome of inappropriate antidiuretic hormone secretion (SIADH) rather than to hypervolemia. Ascitic fluid was submitted for cytological examination, immunoperoxidase staining, and circulating tumor‐DNA profiling (Guardant360) (Tables 1 and 2).

**TABLE 1:** Key diagnostic findings in an 89‐year‐old woman with NSCLC presenting without a detectable primary tumor.

Paracentesis results	Ascitic fluid: white‐cell count 1293 cells/μL with 62% polymorphonuclear neutrophils, indicative of spontaneous bacterial peritonitis; cultures negative
Follow‐up imaging	Bilateral pleural effusions, significant ascites, peritoneal edema, omental caking, and a right adrenal mass (CT of the head, chest, abdomen, and pelvis); no spinal cord compression or metastases (MRI of the brain and spine)
Final cytology and molecular profiling	Positive for Ber‐EP4, EpCAM, and TTF‐1; negative for calretinin, Wilms tumor 1 (WT1), PAX8, and GATA3; mutations in EZH2, TP53, GATA3, and ARID1A (Guardant360)

Abbreviations: CT, computed tomography; MRI, magnetic resonance imaging; NSCLC, non‐small cell lung carcinoma; TTF‐1, thyroid transcription factor‐1; WT1, Wilms tumor 1.

**TABLE 2:** Summary of laboratory and bedside investigations during the hospital course.

Investigation	Result	Reference range/interpretation
Serum sodium (initial presentation)	120 mmol/L	135–145 mmol/L; severe hyponatremia
Serum sodium (after treatment)	123 mmol/L	Partial correction with fluid restriction and sodium chloride
Serum–ascites albumin gradient (SAAG)	0.8 g/dL	< 1.1 g/dL indicates non–portal‐hypertensive (low‐gradient) ascites
Ascitic‐fluid white‐cell count	1293 cells/μL (62% PMNs)	PMN ≥ 250 cells/μL meets the criterion for spontaneous bacterial peritonitis
Ascitic‐fluid culture	No growth	—
Left ventricular ejection fraction (echocardiography)	55%–65%	Preserved systolic function; grade 1 diastolic dysfunction

Abbreviations: PMN, polymorphonuclear neutrophil; SAAG, serum–ascites albumin gradient.

### 3.3 Treatment

Empirical therapy for SBP was started with intravenous ceftriaxone, with metronidazole added for anaerobic coverage, for a total course of 10 days. The hyponatremia was managed with oral sodium chloride tablets and fluid restriction, consistent with SIADH.

## 4 Conclusion and Results (Outcome and Follow‐Up)

Her serum sodium improved to 123 mmol/L and she was discharged. One week later she returned with persistent altered mental status. Re‐examination of the ascitic‐fluid specimen obtained during the index admission revealed malignant cells morphologically compatible with NSCLC. Immunoperoxidase staining and Guardant360 profiling corroborated metastatic carcinoma of pulmonary origin, and repeat cross‐sectional imaging now showed bilateral pleural effusions, significant ascites, peritoneal edema, omental caking, and a right adrenal mass; magnetic resonance imaging of the brain and spine demonstrated no cord compression or central nervous system metastases (Table 1). Despite this thorough imaging survey, no primary pulmonary mass was ever identified, and the lung was established as the tissue of origin on the basis of the immunophenotype (positivity for TTF‐1, Ber‐EP4, and EpCAM).

Following oncology consultation and transfer to a rehabilitation facility, the patient and her family were counseled that systemic anticancer therapy was not appropriate given her advanced age and limited functional reserve, and a palliative, symptom‐focused approach was adopted.

## 5 Discussion

NSCLC is the most common form of lung cancer and comprises a heterogeneous group of histological subtypes. It is classified into three main subtypes—adenocarcinoma (ADC), squamous cell carcinoma (SCC), and large cell carcinoma (LCC)—with several additional rare subtypes recognized. Differences in the pathogenic and genetic features of these subtypes are thought to confer distinct clinical behaviors and treatment responses [11]. The present case illustrates the difficulty of diagnosing NSCLC and of managing patients in whom no conventional primary tumor is identified. The absence of an apparent lung tumor created diagnostic ambiguity and delayed the cancer diagnosis. A definitive diagnosis of NSCLC was reached only after a systematic appraisal of the clinical, radiological, cytological, and molecular findings. Notably, because invasive tissue sampling was not pursued in this frail, elderly patient, the diagnosis rested on ascitic‐fluid cytology and immunocytochemistry rather than on a histological biopsy specimen.

Primary lung cancer frequently metastasizes to the brain, liver, adrenal glands, and bones [2]. In NSCLC, metastases to soft tissue and to the kidney, pancreas, spleen, peritoneum, intestine, bone marrow, eye, ovary, thyroid, heart, breast, tonsil, and nasal cavity have all been reported and generally indicate a poor outcome [2]. Gastrointestinal metastasis from lung carcinoma is rare. In a case series of 1552 patients reported by Fujiwara et al. [12], gastric metastasis was identified in only one patient, and various studies have reported an incidence of between 0.2% and 1.7% [13]. The rarity of such occurrences mandates a high index of suspicion when patients present with atypical symptoms.

NSCLC presenting without a detectable primary tumor is uncommon, and the precise mechanism underlying the absence of a measurable primary lesion remains unknown. Several theories have been proposed, including spontaneous regression of the underlying tumor, rapid growth and early dissemination of the metastatic lesions, or the presence of a primary tumor in an anatomical region not captured by the imaging studies performed [4, 14].

In our patient, the initial clinical manifestations were progressive abdominal distension, decreased oral intake, and altered mental status. After common causes had been excluded, further investigation—including imaging and diagnostic paracentesis—established the diagnosis of NSCLC. Analysis of the ascitic fluid revealed malignant cells morphologically consistent with NSCLC, confirming metastatic disease. Imaging demonstrated small bilateral pleural effusions, omental caking, and a right adrenal mass consistent with metastatic NSCLC [15, 16]. Immunocytochemistry performed on the ascitic‐fluid cell block was central to the diagnosis. The malignant cells were positive for the epithelial markers Ber‐EP4 and EpCAM and for thyroid transcription factor‐1 (TTF‐1), a marker of pulmonary (and thyroid) lineage, supporting a lung primary; they were negative for calretinin and Wilms tumor 1 (WT1), arguing against a mesothelial process, and negative for PAX8 and GATA3, arguing against renal or Müllerian and breast or urothelial origins, respectively [9]. NSCLC without a detectable primary tumor poses unique prognostic and therapeutic challenges [4]. The prognosis is generally poor, with a median survival of less than 1 year [7]. The absence of a primary tumor may reflect a more aggressive disease phenotype with early dissemination of metastatic lesions; consequently, treatment strategies focus on palliative care and symptom management to optimize quality of life [7].

NSCLC exhibits a highly intricate genomic architecture shaped by numerous genetic and epigenetic drivers of carcinogenesis [11]. These tumors harbor a wide range of genetic alterations, with a notable prevalence of somatic mutations, as well as gene copy‐number changes and chromosomal rearrangements that generate oncogenic fusion proteins [11]. Such alterations include amplifications and deletions of key genes regulating chromatin dynamics and the cell cycle, underscoring the broad molecular heterogeneity that drives the initiation and spread of NSCLC [11]. Comprehensive molecular profiling with Guardant360 in our case revealed multiple mutations, including EZH2, TP53, GATA3, and ARID1A. These mutations may inform tumor biology and represent potential targets for novel therapies or enrollment in corresponding clinical trials [17]. The treatment of NSCLC continues to evolve with the integration of multimodal approaches that may include chemotherapy, radiotherapy, tumor‐treating fields (TTFields), targeted therapies, and immunotherapies. Surgery plays an important role in early‐stage NSCLC and, in selected patients, in advanced disease, and ongoing research continues to refine and expand treatment options. Our patient, however, was not a candidate for systemic treatment because her functional status and advanced age precluded its use.

This case report adds to the limited literature on this rare form of NSCLC, which here presented as malignant ascites with no other evident site of metastasis. It also emphasizes the importance of a comprehensive diagnostic evaluation and an individualized treatment approach, particularly in elderly patients with serious illness.

### 5.1 Limitations

This report has several limitations. As a single case, it cannot establish causation or yield generalizable management recommendations. The diagnosis rested on ascitic‐fluid cytology, immunocytochemistry, and circulating tumor‐DNA analysis rather than on a histological tissue‐biopsy specimen, because the patient's age, functional status, and goals of care precluded invasive sampling or resection; definitive histological subtyping of the NSCLC was therefore not obtained, and the pulmonary origin was inferred from the immunomolecular profile rather than demonstrated directly. Despite comprehensive CT of the chest, abdomen, and pelvis and MRI of the brain and spine, no primary lung lesion was localized, and positron‐emission tomography—which might have disclosed an occult primary or additional metastatic foci—was not performed. Finally, because active oncological treatment was not pursued, longer‐term follow‐up and treatment‐response data are not available. These limitations notwithstanding, the case documents a diagnostically challenging and rarely reported presentation of NSCLC.

In conclusion, when NSCLC presents without a visible primary tumor, diagnosis can be difficult and a multidisciplinary, expert approach is required to reach the correct diagnosis and select appropriate treatment. Despite ongoing study, the underlying mechanisms and optimal management of this uncommon manifestation remain incompletely understood, and reliable prognostic indicators and effective therapies are needed to improve outcomes. Our case underscores the importance of giving malignancy a high priority in the differential diagnosis of elderly patients who present with progressive abdominal distension.

## Author Contributions

**Ashish Sharma:** conceptualization, investigation, funding acquisition, writing – original draft, methodology, validation, visualization, writing – review and editing, project administration, formal analysis, software, data curation, resources. **Angad Tiwari:** conceptualization, investigation, funding acquisition, writing – original draft, methodology, validation, visualization, writing – review and editing, software, project administration, formal analysis, data curation, resources. **Monica Lewkowicz:** conceptualization, writing – original draft, writing – review and editing, visualization, validation, supervision. **Hareesha Rishab Bharadwaj:** supervision, conceptualization, writing – original draft, writing – review and editing, visualization, validation. **Edgar Naut:** conceptualization, funding acquisition, writing – original draft, writing – review and editing, visualization, validation, supervision. **Khabab Abbasher Hussien Mohamed Ahmed:** conceptualization, writing – original draft, writing – review and editing, visualization, validation, supervision.

## Funding

The authors have nothing to report.

## Ethics Statement

Institutional review board approval was not required for this single, retrospective, de‐identified case report in accordance with institutional policy.

## Consent

Written informed consent for the publication of this case report and the accompanying image was obtained from the patient's next of kin (legally authorized representative), given the patient's altered mental status at the time of care. The authors confirm that the consent obtained complies with the requirements set out in the journal's author guidelines, and a copy is available for review by the Editor‐in‐Chief on request.

## Conflicts of Interest

The authors declare no conflicts of interest.

## Data Availability

Data sharing not applicable to this article as no datasets were generated or analyzed during the current study.
